# Generative Artificial Intelligence in Patient Education: ChatGPT Takes on Hypertension Questions

**DOI:** 10.7759/cureus.53441

**Published:** 2024-02-02

**Authors:** Ahmed Almagazzachi, Ahmed Mustafa, Ashkan Eighaei Sedeh, Andres E Vazquez Gonzalez, Anastasiia Polianovskaia, Muhanad Abood, Ameer Abdelrahman, Veronica Muyolema Arce, Talar Acob, Bushra Saleem

**Affiliations:** 1 Internal Medicine, Capital Health System, Trenton, USA; 2 Internal Medicine Residency Program, Capital Health Regional Medical Center, Trenton, USA

**Keywords:** cardiology research, general internal medicine, generative ai, ai in cardiology, patient education, hypertension, artificial intelligence in medicine, chatgpt

## Abstract

Introduction

Uncontrolled hypertension significantly contributes to the development and deterioration of various medical conditions, such as myocardial infarction, chronic kidney disease, and cerebrovascular events. Despite being the most common preventable risk factor for all-cause mortality, only a fraction of affected individuals maintain their blood pressure in the desired range. In recent times, there has been a growing reliance on online platforms for medical information. While providing a convenient source of information, differentiating reliable from unreliable information can be daunting for the layperson, and false information can potentially hinder timely diagnosis and management of medical conditions. The surge in accessibility of generative artificial intelligence (GeAI) technology has led to increased use in obtaining health-related information. This has sparked debates among healthcare providers about the potential for misuse and misinformation while recognizing the role of GeAI in improving health literacy. This study aims to investigate the accuracy of AI-generated information specifically related to hypertension. Additionally, it seeks to explore the reproducibility of information provided by GeAI.

Method

A nonhuman-subject qualitative study was devised to evaluate the accuracy of information provided by ChatGPT regarding hypertension and its secondary complications. Frequently asked questions on hypertension were compiled by three study staff, internal medicine residents at an ACGME-accredited program, and then reviewed by a physician experienced in treating hypertension, resulting in a final set of 100 questions. Each question was posed to ChatGPT three times, once by each study staff, and the majority response was then assessed against the recommended guidelines. A board-certified internal medicine physician with over eight years of experience further reviewed the responses and categorized them into two classes based on their clinical appropriateness: appropriate (in line with clinical recommendations) and inappropriate (containing errors). Descriptive statistical analysis was employed to assess ChatGPT responses for accuracy and reproducibility.

Result

Initially, a pool of 130 questions was gathered, of which a final set of 100 questions was selected for the purpose of this study. When assessed against acceptable standard responses, ChatGPT responses were found to be appropriate in 92.5% of cases and inappropriate in 7.5%. Furthermore, ChatGPT had a reproducibility score of 93%, meaning that it could consistently reproduce answers that conveyed similar meanings across multiple runs.

Conclusion

ChatGPT showcased commendable accuracy in addressing commonly asked questions about hypertension. These results underscore the potential of GeAI in providing valuable information to patients. However, continued research and refinement are essential to evaluate further the reliability and broader applicability of ChatGPT within the medical field.

## Introduction

Hypertension is a global epidemic affecting 31.1% of the adult population worldwide and is often regarded as the “silent killer” due to its contribution to the onset and exacerbation of major debilitating conditions such as myocardial infarction, chronic kidney disease, and stroke [1,2]. As the most significant preventable risk factor for all-cause mortality, the management of hypertension is crucial; yet, only a mere 21% of those affected have their blood pressure optimally controlled [2]. Optimizing hypertension control could lead to a 49% decrease in cardiac mortality and a significant 62% reduction in cerebrovascular mortality [3]. In recent years, with technological advancements and the increased accessibility and availability of online resources, there has been a noticeable surge in reliance on online platforms as a source of medical information [4]. However, accessing online resources for medical information can be a double-edged sword. While offering a convenient gateway to health-related information at the click of a button, the availability of unreliable and false information can hinder patient care and pose a potential threat to timely diagnosis and prompt management of medical conditions [4]. The emergence of generative artificial intelligence (GeAI) technology, coupled with its widespread availability to the public, has ushered in a new era of acquiring medical information. This shift has sparked conflicting opinions within the healthcare community, navigating the delicate balance between acknowledging the constructive role that GeAI can play in enhancing health literacy while also recognizing the potential for misuse and misinformation [4].

## Materials and methods

In this nonhuman-subject qualitative study, the accuracy and reproducibility of responses offered by GeAI on hypertension questions were assessed. ChatGPT, developed by OpenAI in 2024, was selected as the GeAI of choice due to its popularity and accessibility for public use. Three internal medicine residents enrolled in an ACGME-accredited program gathered questions commonly asked by patients on the subject of hypertension, including the risk factors associated with having high blood pressure, management of hypertension, and complications of uncontrolled blood pressure. To ensure the validity and relevance of the selected questions, a board-certified internal medicine physician with over eight years of experience in diagnosing and managing hypertension evaluated each question, eliminating questions deemed less relevant, and finalized the question list used for this study. Each question then underwent three separate runs through ChatGPT, resulting in three independent sets of responses to each question. Responses generated by ChatGPT were first assessed for reproducibility. For a response to be deemed reproducible, all three answers to the same question needed to convey the same message. Any variance in the message resulted in the classification of that corresponding response as non-reproducible. Following the assessment of reproducibility, the study delved into evaluating response accuracy. To evaluate the accuracy of a response, it was checked against the recommended guidelines from reputed resources such as the American Heart Association or the National Institute of Health. The majority response to each question was first selected; an answer repeated at least two out of three times was selected as the majority response and compared against the recommended guidelines. Responses were categorized as appropriate if they aligned with the guidelines or inappropriate if they deviated. As a final step, a board-certified internal medicine physician conducted a final review of each question and answer and evaluated the appropriateness of responses from a clinical standpoint. For the assessment of response accuracy, therefore, each response had two sets of evaluations, one based on the recommended guidelines and one based on the clinical opinion of the board-certified physician. An overall accuracy score was calculated by averaging the accuracy score obtained from comparing the responses versus guidelines as well as the evaluation of responses by the physician. Descriptive statistics were used to examine the accuracy and reproducibility of the responses generated by ChatGPT.

## Results

**Figure 1:**
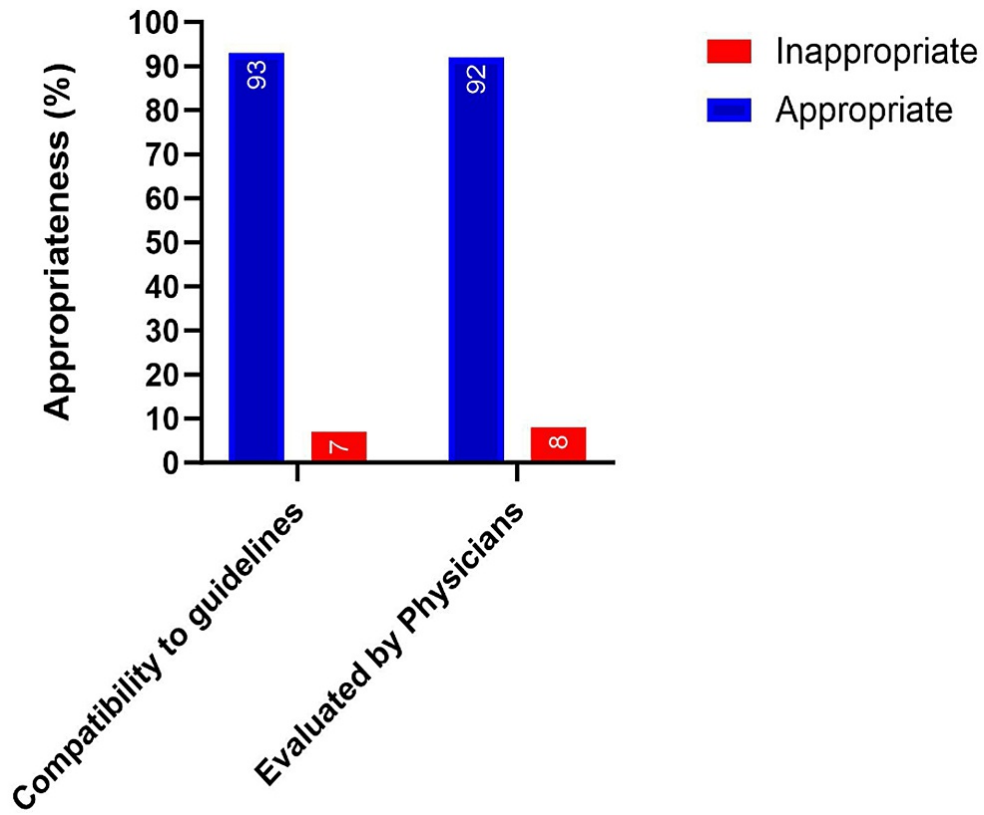
Appropriateness of responses provided by ChatGPT to hypertension-related queries

**Figure 2:**
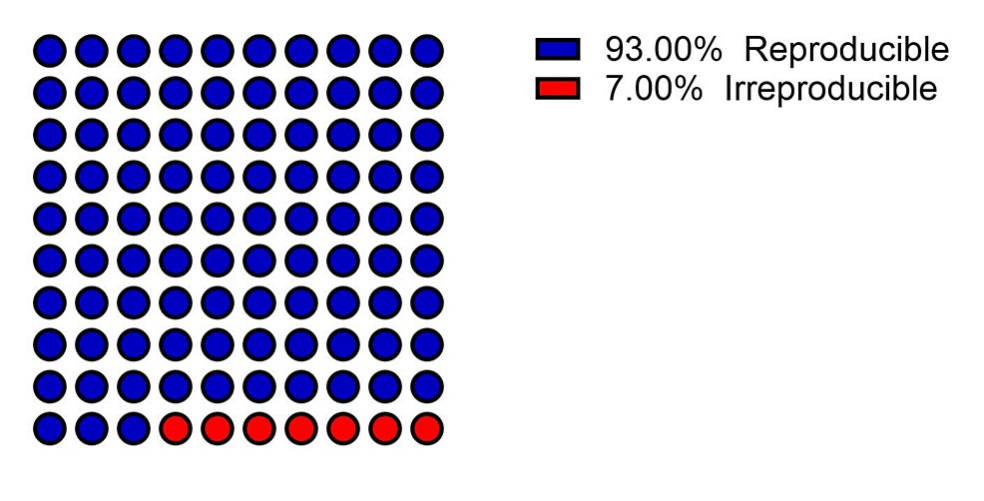
Reproducibility of ChatGPT responses

Initially, 130 frequently asked questions on the subject of hypertension were gathered by three internal medicine residents enrolled in an ACGME-accredited program. These questions were then revised for relevance by a board-certified internal medicine attending physician with over 8 years of experience in diagnosing and managing hypertension, resulting in a final set of 100 questions selected for the purpose of this study (Table 1). ChatGPT responses were assessed against recommended guidelines and exhibited robust accuracy with an appropriateness rate of 93%, while 7% of responses were deemed inappropriate. A parallel evaluation by a board-certified physician revealed a similar degree of appropriateness of the responses offered by ChatGPT, with 92% of the responses being classified as appropriate, while 8% were inappropriate (Figure 1). Therefore, averaging the accuracy of the responses by ChatGPT resulted in an overall accuracy of 92.5%. In the reproducibility evaluation, each question underwent three ChatGPT runs, generating three sets of responses for each question and totaling 300 responses. Analysis of these 300 responses revealed that ChatGPT provided irreproducible answers in 7 instances (3.6% of the total responses). However, when calculating reproducibility per question, it was found that 93% of the questions had reproducible responses while 7% of the questions had irreproducible responses, highlighting instances where responses could not be consistently replicated across multiple ChatGPT runs (Figure 2).

**Table 1:** Evaluation of ChatGPT responses to questions about hypertension BP: Blood pressure; ER: Emergency room; EKG: Electrocardiogram; CT scan: Computed tomography scan.

The selected 100 questions	Accuracy of the responses compared against National Guidelines	Answer evaluated by Board-certified IM Physician	Guidelines
1. Does yoga help to reduce my BP?	Appropriate	Appropriate	Chauhan et al. (2017) [5]
2. What is the normal range of BP for a 65 and older?	Inappropriate	Inappropriate	Whelton et al. (2017) [6]
3. What is considered high BP for men?	Appropriate	Appropriate	Whelton et al. (2017) [6]
4. What is considered high BP for women?	Appropriate	Appropriate	Whelton et al. (2017) [6]
5. What is considered high BP for Caucasian people?	Appropriate	Appropriate	Whelton et al. (2017) [6]
6. What is considered high BP for black African Americans?	Appropriate	Appropriate	Whelton et al. (2017) [6]
7. What is considered high BP for Latinos and Hispanics?	Appropriate	Appropriate	Whelton et al. (2017) [6]
8. What is considered high BP for Asian people?	Appropriate	Appropriate	Whelton et al. (2017) [6]
9. What is considered high BP for Middle Eastern people?	Appropriate	Appropriate	Whelton et al. (2017) [6]
10. What is considered high BP for native Americans?	Appropriate	Appropriate	Whelton et al. (2017) [6]
11. What range of BP should I maintain if I was diagnosed with hypertension?	Appropriate	Appropriate	Whelton et al. (2017) [6]
12. What range of BP should I maintain if I was diagnosed with coronary artery disease?	Appropriate	Appropriate	Whelton et al. (2017) [6]
13. What range of BP should I maintain if I was diagnosed with diabetes mellitus type 2?	Inappropriate	Inappropriate	Whelton et al. (2017) [6]
14. What range of BP should I maintain if I was diagnosed with chronic kidney disease?	Inappropriate	Inappropriate	Whelton et al. (2017) [6]
15. What range of BP should I maintain if I had a stroke in the past?	Appropriate	Appropriate	Whelton et al. (2017) [6]
16. Is a one-time elevation in my BP enough for a diagnosis of hypertension?	Appropriate	Appropriate	Whelton et al. (2017) [6]
17. Does the time of the day matter when I check my BP?	Appropriate	Appropriate	Whelton et al. (2017) [6]
18. Can I have hypertension if I do not feel any symptoms?	Appropriate	Appropriate	Hypertension (2023) [2]
19. Can hypertension cause headaches?	Appropriate	Appropriate	Hypertension (2023) [2]
20. Can hypertension cause blurred vision?	Appropriate	Appropriate	Hypertension (2023) [2]
21. Can hypertension cause shortness of breath?	Appropriate	Appropriate	Hypertension (2023) [2]
22. Can hypertension cause chest pain?	Appropriate	Appropriate	Hypertension (2023) [2]
23. Can hypertension cause abdominal pain?	Appropriate	Appropriate	Hypertension (2023) [2]
24. Can hypertension cause nausea and vomiting?	Appropriate	Appropriate	Hypertension (2023) [2]
25. Is it safe to consume alcohol if I have hypertension?	Appropriate	Appropriate	Whelton et al. (2017) [6]
26. How much caffeine I can consume if I have hypertension?	Inappropriate	Inappropriate	Whelton et al. (2017) [6]
27. Can I (lower / control) my BP with exercise?	Appropriate	Appropriate	Whelton et al. (2017) [6]
28. How long do I need to exercise per week to reduce my BP?	Appropriate	Appropriate	Whelton et al. (2017) [6]
29. Can I (lower / control) my BP with diet changes?	Appropriate	Appropriate	Whelton et al. (2017) [6]
30. Can I (lower / control) my BP with weight loss?	Appropriate	Appropriate	Whelton et al. (2017) [6]
31. Can tobacco smoking increase my BP?	Appropriate	Appropriate	Whelton et al. (2017) [6]
32. Can smoking cessation reduce my BP?	Appropriate	Appropriate	Hypertension (2023), Whelton et al. (2017) [2,6]
33. Does stress affect my BP / my hypertension?	Appropriate	Appropriate	Hypertension (2023) [2]
34. Can a standing desk improve my hypertension?	Appropriate	Appropriate	Hypertension (2023) [2]
35. Can my diabetes cause hypertension?	Appropriate	Appropriate	Hypertension (2023) [2]
36. Can my heart disease cause hypertension?	Appropriate	Appropriate	Whelton et al. (2017) [6]
37. Can cigarette smoking cause hypertension?	Appropriate	Appropriate	Whelton et al. (2017) [6]
38. Can kidney disease cause hypertension?	Appropriate	Appropriate	Whelton et al. (2017) [6]
39. Can I do scuba diving if I have hypertension?	Appropriate	Appropriate	Westerweel et al. (2020) [7]
40. Can high cholesterol cause hypertension?	Appropriate	Appropriate	High Cholesterol (2022) [8]
41. Can vitamin D deficiency cause hypertension?	Appropriate	Appropriate	Ullah et al. (2009) [9]
42. Can COVID cause hypertension?	Appropriate	Appropriate	Akpek et al. (2021) [10]
43. Will I have a normal life expectancy if I take my hypertension medications?	Appropriate	Appropriate	Hypertension (2023), Whelton et al. (2017) [2,6]
44. Can hypertension reduce life expectancy?	Appropriate	Appropriate	Whelton et al. (2017) [6]
45. Can hypertension affect my sex life?	Appropriate	Appropriate	Ferrario et al. (2002) [11]
46. Can hypertension affect my kidneys?	Appropriate	Appropriate	Whelton et al. (2017) [6]
47. Can hypertension affect my heart?	Appropriate	Appropriate	Whelton et al. (2017) [6]
48. Can hypertension affect my heart failure?	Appropriate	Appropriate	Whelton et al. (2017) [6]
49. Can hypertension affect my eyes or my vision?	Appropriate	Appropriate	Bhargava et al. (2011) [12]
50. Can hypertension affect my diabetes?	Appropriate	Appropriate	Hypertension (2023), Whelton et al. (2017) [2,6]
51. Do long-haul flights have an impact on my BP	Appropriate	Appropriate	Okyay et al. (2021) [13]
52. Can I get a stroke because of my hypertension?	Appropriate	Appropriate	Whelton et al. (2017) [6]
53. Can my hypertension cause cancer?	Appropriate	Appropriate	Han et al. (2017) [14]
54. Can I take Viagra if I have hypertension?	Appropriate	Appropriate	Vardi et al. (2002) [15]
55. What can happen if I don’t take my blood pressure-lowering medications?	Appropriate	Appropriate	Whelton et al. (2017) [6]
56. If I have hypertension, are my kids at risk of developing hypertension too?	Appropriate	Appropriate	Dodoo et al. (2017) [6]
57. How often do I need to check my BP if I do not have hypertension?	Appropriate	Appropriate	Whelton et al. (2017) [6]
58. How often do I need to check my BP if I have hypertension?	Inappropriate	Inappropriate	Whelton et al. (2017) [6]
59. How often do I need to check my BP if I have diabetes?	Appropriate	Inappropriate	Your Diabetes Care Schedule (2023) [16]
60. How often do I need to check my BP if I have a Previous stroke?	Inappropriate	Inappropriate	Castilla-Guerra et al. (2015) [17]
61. Is my BP accurate if I just walk upstairs/exercise?	Appropriate	Appropriate	Whelton et al. (2017) [6]
62. How long do I need to sit/rest before checking my BP to get an accurate reading?	Appropriate	Appropriate	Whelton et al. (2017) [6]
63. Is there any difference between right and left arm BP readings?	Appropriate	Appropriate	Fred et al. (2013) [18]
64. What is the best position for a BP check?	Appropriate	Appropriate	Whelton et al. (2017) [6]
65. Is hypertension curable?	Appropriate	Appropriate	Lewanczuk et al. (2008) [19]
66. Is hypertension a life-long disease?	Appropriate	Appropriate	Lewanczuk et al. (2008) [19]
67. If I was prescribed an antihypertensive medication do I need to continue taking it for the rest of my life?	Appropriate	Appropriate	Van der Wardt et al. (2017) [20]
68. Where do I need to go if I find that I have high BP?	Appropriate	Appropriate	High Blood Pressure (2022) [21]
69. Does uncontrolled hypertension increase the chance of bleeding in my brain?	Appropriate	Appropriate	Whelton et al. (2017) [6]
70. If have hypertension and I feel a severe headache what should I do?	Appropriate	Appropriate	High Blood Pressure (2022) [21]
71. When do I need to visit the ER with high BP?	Appropriate	Appropriate	High Blood Pressure (2022) [21]
72. If I have uncontrolled hypertension and I feel dizzy what should I do?	Appropriate	Appropriate	High Blood Pressure (2022) [21]
73. If I have uncontrolled hypertension and I feel chest pain what should I do?	Appropriate	Appropriate	High Blood Pressure (2022) [21]
74. If I have uncontrolled hypertension and I feel short of breath what should I do?	Appropriate	Appropriate	High Blood Pressure (2022) [21]
75. If I have uncontrolled hypertension and I feel abdominal pain what should I do?	Appropriate	Appropriate	High Blood Pressure (2022) [21]
76. If I have uncontrolled hypertension and I feel a limb weakness what should I do?	Appropriate	Appropriate	High Blood Pressure (2022) [21]
77. If I have hypertension and I get red eyes is it dangerous?	Appropriate	Appropriate	Tarlan et al. (2013) [22]
78. If I have a new diagnosis of hypertension do I need an EKG?	Appropriate	Appropriate	Whelton et al. (2017) [6]
79. If I have a new diagnosis of hypertension do I need a urine test?	Appropriate	Appropriate	Whelton et al. (2017) [6]
80. If I have a new diagnosis of hypertension do I need a kidney function test?	Appropriate	Appropriate	Whelton et al. (2017) [6]
81. If I have a new diagnosis of hypertension do I need a liver function test?	Appropriate	Appropriate	Whelton et al. (2017) [6]
82. If I have a new diagnosis of hypertension do I need an Echocardiogram?	Appropriate	Appropriate	Whelton et al. (2017) [6]
83. Do I need a blood test to diagnose hypertension?	Appropriate	Appropriate	Whelton et al. (2017) [6]
84. If I have a new diagnosis of hypertension do I need a serum electrolytes test?	Appropriate	Appropriate	Whelton et al. (2017) [6]
85. If I have a new diagnosis of hypertension do I need a CT scan of my brain?	Appropriate	Appropriate	Kraniotis et al. (2015) [23]
86. If I have a new diagnosis of hypertension do I need a chest X-ray?	Appropriate	Appropriate	Mirsadraee et al. (2013) [24]
87. What is the best treatment for hypertension?	Appropriate	Appropriate	Whelton et al. (2017) [6]
88. Do I need surgery for my hypertension?	Appropriate	Appropriate	Saha et al. (2015) [25]
89. What food helps to prevent hypertension?	Appropriate	Appropriate	Whelton et al. (2017) [6]
90. Does hypertension increase the risk of getting an abdominal aortic aneurysm?	Appropriate	Appropriate	Whelton et al. (2017) [6]
91. Is hypertension a contraindication for taking aspirin?	Appropriate	Appropriate	Bautista et al. (2010) [26]
92. Is hypertension a contraindication for taking ibuprofen?	Appropriate	Appropriate	Bautista et al. (2010) [26]
93. Is hypertension a contraindication for taking Tylenol?	Appropriate	Appropriate	MacIntyre et al. (2022) [27]
94. Can hypertension affect my pregnancy?	Appropriate	Appropriate	Preeclampsia (2022) [6]
95. Can I eat fast food if I have hypertension?	Appropriate	Appropriate	Hypertension (2023) [2]
96. Can hypertension be passed to others?	Appropriate	Appropriate	Dodoo et al. (2017) [6]
97. What is the average age for developing hypertension?	Appropriate	Appropriate	Whelton et al. (2017) [6]
98. Can cancer (type of cancer) cause hypertension?	Appropriate	Appropriate	Whelton et al. (2017) [6]
99. What is the normal range of BP for a 50-year-old?	Inappropriate	Inappropriate	Whelton et al. (2017) [6]
100. Can I lift if I have hypertension?	Appropriate	Appropriate	Hypertension (2023) [2]

## Discussion

This study aligns with the continuous endeavor to assess the precision of health-related content offered by GeAI [4]. These efforts are imperative; identifying reliable online information sources can help with health literacy while limiting medical misinformation. The significance of misinformation in healthcare has been demonstrated by Bremner et al. in a study focused on psychological trauma, where it was found that only 42% of the information obtained by patients through online search engines to be accurate [28]. Particularly in the medical field, even slight misinformation can contribute to misconceptions, inadvertently influencing medical decisions [29]. Due to the novelty of GeAI as a patient education tool, there needs to be more direct comparisons in the literature. Nonetheless, this study's findings align with other studies that explored the potential role that GeAI can play in advancing health literacy [4]. While the information provided by GeAI is compatible with current practices, it lacks the human touch. Though this does not impact the quality of the information provided, this could, in fact, affect patients' utilization and reliance on GeAI. This is particularly true when patients are at their most vulnerable, for example, when learning about a chronic medical condition such as hypertension, to the point that up to 60% of Americans do not feel comfortable relying on AI for health-related information. [30]. There were limitations to the current study. First, the evaluation was based on a predefined set of questions, which may cover only some hypertension-related topics. Furthermore, it is worth noting that the questions posed to ChatGPT were exclusively in English, and employing a different language could yield varied results. Additionally, the study focused on qualitative assessment, and user interactions and context were not considered, which may affect the quality of responses. Furthermore, the ChatGPT responses were assessed by staff who are trained clinicians, and further studies have to be performed, perhaps those including patient subjects, to determine the receptive quality of the information offered by GeAI from a patient’s standpoint, including but not limited to, presence of jargon, and the clarity of the language.

## Conclusions

This study emphasizes the potential of AI-generated information in providing appropriate and readily accessible medical knowledge, serving as a potential patient educational tool in the near future. However, overall accuracy and the lack of complete consistency remain questionable areas where GeAI, such as ChatGPT, needs improvement before being readily used for a sensitive purpose like health education. While GeAI as an accessible resource for health literacy may eventually facilitate increased involvement of patients in their care and ultimately improve compliance and long-term outcomes in chronic disorders, continued research on the subject of GeAI in healthcare is critical to further validate the accuracy and reproducibility of AI-generated health information which can potentially allow for generalizability of the role of GeAI in health literacy. Lastly, considering the sensitivity of health-related information and the importance of accurate information, it is recommended that GeAI be complemented by human supervision and oversight to ensure accurate and reliable information is offered.
